# Evaluation of the Prevalence of Urinary Tract Infection in Rural Panamanian Women

**DOI:** 10.1371/journal.pone.0047752

**Published:** 2012-10-19

**Authors:** Suzanne L. August, Michael J. De Rosa

**Affiliations:** 1 School of Nursing, Samuel Merritt University, Oakland, California, United States of America; 2 Physician Assistant Department, Samuel Merritt University, Oakland, California, United States of America; Vanderbilt University, United States of America

## Abstract

**Objective:**

Urinary tract infection (UTI) is the most common non-intestinal infection worldwide. In the developed world, incidence and prevalence of UTI would be similar owing to the relatively short duration of illness experienced by women with ready access to healthcare services. We hypothesize that, in the developing world, factors limiting access to care and those which may increase the likelihood of developing UTI, result in increased morbidity. This difference is reflected in an increased prevalence of UTI in regions where women suffer the effects of UTI for extended periods of time.

**Methods:**

This study represents a cross sectional analysis of UTI prevalence in rural western Panama conducted over the course of a 3-day medical mission. All women 18–45 years of age reporting to the medical brigade clinic were tested for UTI by dipstick urinalysis and a brief history regardless of whether they themselves were presenting with a complaint.

**Results:**

UTI was diagnosed clinically by providers in 29.8% of the women tested although only 21.15% of these same women met the evidence-based study criteria. This prevalence of 21.15% is seven times greater than reported by the Panamanian Ministry of Health. When comparing the effectiveness of clinical diagnosis relative to urinalysis by dipstick, a Kappa coefficient revealed only low moderate agreement (0.42; SE 0.0955).

**Conclusions:**

The prevalence of UTI in rural western Panama is greater than would be expected based on prevalence data from either the US or Panamanian Ministry of Health and may represent an opportunity for targeted interventions, including educational programming about UTI prevention.

## Introduction

This study represents a cross sectional analysis of the prevalence of urinary tract infections (UTI) among women of reproductive age in two rural communities of western Panama. Previously, we observed an unusually high occurrence of hematuria, pyuria, UTI and pyelonephritis in the adult female population of Chiriqui, the westernmost province of Panama. It was noted that women complained of these symptoms for periods ranging from several days to as long as four years. While, very little information is available on the incidence and prevalence of UTI in Panama or any other Central American country, Ministry of Health and published data suggest that UTI is a significant source of morbidity in this population. On a return trip to the neighboring province of Veraguas, we were able to more carefully document urinary tract infection rates in that community. As a result of our informal findings on previous trips and our inability to conduct confirmatory urine cultures, a medical decision was made to test all females of reproductive age by dipstick urinalysis allowing for the study of UTI in women presenting to the clinic. The population studied consists largely of *campasinas*, rural poor who are mostly day laborers and subsistence farmers. Their access to health care is limited by distance and the lack of transportation or financial resources.

Urinary tract infection is the most common non-intestinal infection in women worldwide [1]. The pathogens causing UTI are consistent across the globe. The pathogenesis of urinary tract infection involves ascending infection with coliform bacteria colonizing the perineum in susceptible women (80–90% *Escherichia coli*, 5–10% *Staphylococcus saprophyticus* with the remainder caused by *Proteus* and other Gram negative rods) [2]. While not generally considered a cause of significant mortality, UTI do represent an important cause of morbidity. Typical symptoms associated with UTI include the triad of dysuria (painful urination), urgency (the enhanced desire to void the bladder) and frequency (increased frequency of urination). While not thought to cause significant mortality, UTI have the potential for serious and life-threatening sequelae if left untreated or undertreated. This is more likely to be the case where access to or availability of timely and appropriate medical intervention is limited due to inadequate numbers of health care providers such as in much of Central America. Possible sequelae include pyelonephritis which can lead to renal scarring and sepsis [3]. UTI can be particularly dangerous in pregnant women in whom it has been shown that up to 50% of those with asymptomatic bacteriuria (ABU) go on to develop pyelonephritis. In addition, these women experience higher rates of intrauterine growth restriction and low birth-weight infants. The presence of a UTI has also been shown to increase the risk of preterm labor, preterm birth, pregnancy-induced hypertension, preeclampsia, amnionitis and anemia [4].

There are also public health issues associated with UTI related to quality of life. Since 1948, the World Health Organization (WHO) has defined health as not just disability or the absence of morbidity but as “complete physical, mental and social well-being” [5]. Lower urinary tract symptoms which accompany UTI include bothersome sensations such as urinary urgency, frequency, painful urination, hesitancy, and the sense of incomplete bladder emptying. Liao [6] examined the effects of such symptoms on the quality of life for 907 nurses in Taipei. The findings indicated a negative impact on health related to the poor quality of life caused by these symptoms [6]. In addition to a reduction in quality of life for women who are symptomatic, in countries with limited health care dollars and resources, unnecessary UTIs might be expected to cause a drain on the already struggling health care apparatus.

In the United States, UTI account for 8.3 million out-patient visits and 1 million hospitalizations annually [7]. The prevalence of ABU, a reliable predictor of UTI, ranges from 5%–6% in women 18–40 years of age up to 20% in the ambulatory elderly [8]. Most of these UTI are uncomplicated and managed on an outpatient basis with simple antibiotic regimens. In low and middle income countries with reduced access to medical care, UTI may be expected to cause more morbidity and incur greater risk of adverse outcomes when women are unable to get appropriate treatments early in the course of disease.

UTI are likely to be more common in lower income countries than in the US. The majority of the reports in the literature pertaining to urinary tract infections globally have measured ABU in pregnant women. There were no studies found measuring prevalence of UTI or ABU from Latin America specifically, however there were several from other low income countries. Two recent studies in Nigeria have reported prevalence rates of ABU in pregnant women of between 10.7% [9] and 21% [10]. Masinde [11] published a cross-sectional study from Tanzania in 2009 to determine the prevalence of UTI in both symptomatic and asymptomatic pregnant women. Of the 247 women in the sample, 31.5% were symptomatic and of those women, 18% had bacteriuria. Interestingly, of the 68.5% who were asymptomatic, 13% had bacteriuria as well [10]. A study from Bangkok, Thailand [12] reported a prevalence rate for ABU in pregnant women of 10% with an increased risk of ABU in women with a lower educational level. This was consistent with other studies that showed a similar increased risk with either lower educational level or lower socioeconomic level [9], [12], [13].

In 2005, the Panamanian Ministry of Health reported 1379 cases of UTI in the province of Veraguas in the 20–59 year age group, making UTI one of 5 principal causes of morbidity in the report [14]. Just under half of the population of the province (47.2%) is represented by this age group. If we assume that the UTI reported in 2005 were all found in female residents, we would still calculate a regional prevalence of less than 3% from the figure in this report. It is difficult to determine the actual prevalence of UTI in the population from these data; however, we believe, based on our clinical observations, the prevalence of UTI in rural western Panama is likely to be significantly higher than what has been previously reported. Underrepresentation of rural poor outside the major city of Santiago may have contributed to this under-estimation.

## Methods

This study was exempted from review by the Samuel Merritt University Institutional Review Board, because it does not involve human subjects, but rather a *post hoc* review of clinical data without patient-identifying information.

### Population

This study was conducted on three days of medical brigades in the western Panamanian province of Veraguas in collaboration with Global Medical Brigades in April, 2010. Medical clinics were conducted in the rural towns of Ponuga and Marieta in Veraguas province in western Panama. The population seen consisted mostly of rural poor subsistence farmers and laborers known as *campesinas*. On each of the three days, nursing and advanced practice nursing students and licensed faculty from nursing, nurse practitioner and physician assistant departments worked in conjunction with local ministry of health physicians and community leaders to provide primary health care to the people of Veraguas province. Clinics were established in schools and existing health centers in these rural communities where regular access to health care is challenging. Data was collected from routine urinalyses collected during these encounters.

### Data Collection

Based on our previous clinical observations and an understanding of the role of asymptomatic bacteriuria in UTI, we made the *a priori* clinical decision to collect urine specimens for dipstick analysis from all women ages 18–49 presenting to the clinics. Nursing students provided instruction in Spanish for clean catch, mid-stream urine specimens and conducted urinalyses by dipstick testing as part of their routine workup. Clinical encounters were then conducted by Panamanian Ministry of Health physicians and nurse practitioner students. This provided the opportunity to assess UTI prevalence in women seeking care. Nursing students trained to triage patients were instructed to record clinical data, including urinalysis results and relevant symptoms on colored data sheets referred to as “Pink Sheets” which were collected at the end of the mission for data analysis. Study data was collected by retrospective review of the “Pink Sheets” for all women age 18–49 regardless of presentation or chief complaint.

### Definition of UTI

For the purposes of this study, positive UTI was defined as the presence of two of the following three criteria on presentation or urinalysis: leukocyte esterase, nitrites or a documented complaint of dysuria [15]. These criteria are consistent with clinical findings associated with a high predictive value for UTI with each additional criterion making UTI more likely [16]. Data from these clinical indicators were also compared to clinical diagnoses of UTI including pyelonephritis by the clinical team.

## Results

Of 104 women between 18–45 years of age (average age = 31 years) seen on three days of medical brigades in Veraguas, Panama, 31 (29.8%) were diagnosed clinically by brigade providers with either a UTI or pyelonephritis and treated. When the data from pink sheets were analyzed, we found that 22 (21.15%) of the women met the study criteria for UTI and of those that met the criteria, 15 were also diagnosed clinically (Table 1).

**Table 1:** Prevalence of UTI in women 18–45 years of age in Veraguas, Panama.

Category	N	Percentage
**Women 18–45**	104	100
**Diagnosed with UTI**	31	29.80
**Met UTI Criteria**	22	21.15

Analysis of the 22 patients who met study criteria for UTI (Table 2) demonstrates that 13 (59.10%) had a complaint of dysuria with leukocyte esterase (LE) on dipstick urinalysis. Seven of the 22 (31.82%) women in this group had LE and nitrites on dipstick without dysuria. Only one patient had dysuria with nitrites and no evidence of LE on dip while one additional woman was positive for all three criteria.

**Table 2:** Data from analysis of UA results, Veraguas, Panama, April 2010.

UTI positive by study Criteria	N	Percentage	Probability of UTI
**Total**	22	100	
**Dysuria + LE**	13	59.10	.784
**LE + Nitrite**	7	31.82	.727
**Dysuria + Nitrite**	1	0.96	.706
**LE + Nitrite + Dysuria**	1	0.96	.950

Table 3 represents a comparison of patients who met study criteria for UTI and those diagnosed clinically with UTI by the providers on the brigades. Fifteen of the 22 patients (68.18%) who met the study criteria for UTI were diagnosed and treated for UTI. The clinicians on the brigade diagnosed an additional 16 patients who did not meet the criteria for this study. Therefore, on this medical mission the sensitivity of clinical diagnosis was 48.4%. Of the 73 women who were not diagnosed clinically with UTI, 7 met the study criteria representing a specificity of 90.4%. A Kappa coefficient of 0.42 (standard error 0.0955) was calculated to assess the inter-observer variation between clinicians diagnosing UTI during the medical brigade. This was consistent with a low moderate agreement level between clinicians. In all, 31 (29.81%) women were diagnosed with UTI, including two cases of pyelonephritis. Further analysis of this data demonstrates a Risk Ratio of 3.49 (CI: 2.07–5.90; p<.001) for those women found to have two of three study criteria.

**Table 3:** Comparison of UTI diagnosis based on study criteria vs clinical diagnosis by health care providers on the brigades.

		Clinical UTI Diagnosis		
		Positive	Negative	Total
**2 of 3 Study Criteria**	Positive	15	7	22
	Negative	16	66	82
	Total	31	73	104

A more detailed analysis of those patients who met the study criteria for UTI but failed to be diagnosed in the clinical setting (Table 4) demonstrates that five of the seven presented with dysuria. The remaining two had dipstick urinalyses positive for leukocyte esterase and nitrites but reported no dysuria. Stratification of the data for age demonstrated no significant differences in prevalence.

**Table 4:** Detailed analysis of seven women positive for UTI based on study criteria but not diagnosed on site.

UTI study criteria	N
**Leukocyte esterase + dysuria**	5
**Leukocyte + nitrites**	2
Total	7

## Discussion

Infections of the urinary tract (UTI) are the second most common infectious disease in women after gastrointestinal disorders. In the developed world with ready access to health care and antibiotic therapy, UTI tends to be cured very quickly, limiting the morbidity associated with the condition. Additionally, the short time that women spend afflicted with UTI means that, in the developed world, there is little to no difference between the incidence of UTI and its prevalence as very few patients live with the condition chronically. In our experiences in rural Panama where access to care and available resources are much more limited, however, we have noted that many women report durations of symptoms consistent with UTI lasting several years. In low or middle income country, then, one might expect a high prevalence of this condition if the lack of treatment results in women living with UTI over long periods of time. In this study, 22 of 104 (21.15%) women aged 18–45 years met the criteria for UTI. This finding is very similar to the prevalence demonstrated in Nigerian women by Akinloye et al [10] and is consistent with Panamanian Ministry of Health concern regarding UTI as a significant cause of morbidity in the region [14].

In a study of this nature, a potential limitation is found in the reliability of the diagnostic criteria established for making the diagnosis of UTI. Due to our inability to conduct confirmatory microbial cultures to assess our diagnostic criteria, we based our criteria on standard of care for diagnosing UTI in the United States [15], [16]. Medino-Bombardo et al. [16] demonstrated that dysuria had the highest likelihood ratio of the symptoms they studied^15^. In their study positive findings of nitrites or leukocyte esterase as an indicator of pyuria, increased the probability of UTI by 5 and 1.5 fold, respectively, while the presence of both findings increased probability of UTI by 7 fold. We believe findings from the current study are consistent with Medino-Bombardo et al [16] in that the individual study criteria, dysuria, leukocyte esterase and nitrites demonstrated probabilities for UTI of,314,.337 and.252, respectively, while the combinations of these findings we associated with probabilities of UTI of greater than 70% (see Table 2). Additionally, patients in the current study who were positive for two of the three study criteria were 3.5 times more likely to have been diagnosed with a UTI, a finding similar to those of Medino-Bombardo, et al [16].

There was significant discrepancy in this study between the patients identified as suffering from UTI based on our diagnostic criteria and those actually diagnosed by the health care providers on scene. Only 48% of those actually diagnosed with UTI met the criteria for diagnosis for this study. Additionally, nearly one third (7/22) of those meeting our criteria failed to be diagnosed with UTI by the providers on scene. The low moderate Kappa coefficient demonstrates poor agreement between diagnoses made based on clinical criteria and the clinical diagnoses made in the field which is also consistent with published reports. A number of potential explanations for this discrepancy exist, including language difficulties between patients and providers from the US conducting a medical mission, differences in diagnostic priorities among Panamanian vs. American providers, and the presence of asymptomatic bacteriuria resulting in lower diagnosis rates. Asymptomatic bacteriuria does not appear to be the reason for this discrepancy, however, as 5 of the 7 patients who met the UTI criteria but failed to be diagnosed were not asymptomatic, i.e. they reported dysuria. We are unable to determine, using a blinded data set, whether the patients reporting dysuria to the members of our brigades who sampled and tested their urine subsequently denied dysuria when seen by the providers participating in the clinic. The remaining 2 of 7 undiagnosed patients are likely to have had asymptomatic bacteriuria as their urines were positive for leukocyte esterase and nitrites but they denied dysuria. In a retrospective study of hospitalized patients, there was significant difference in UTI diagnoses made by two expert physicians reviewing the same patients’ charts demonstrating the lack of reliability in UTI diagnoses even between experts using predetermined diagnostic criteria [17]. It seems realistic, then, to expect a certain degree of variability in diagnoses with multiple providers from vastly different backgrounds working in unfamiliar and challenging circumstances. Clinical diagnosis of UTI demonstrated a sensitivity of less than 50% in this study.

Other limitations of this study include possible selection bias. As the 3-day trips are billed as “medical brigades” and establish a medical clinic within the community, we may only draw patients from those community members with medical complaints. Healthy women, therefore, may not be included in the patient pool if they fail to report to the clinic to be seen. The failure to include healthy women from the community may exaggerate our findings. To limit the effect of the selection bias, we included all women within our target age group regardless of whether they presented with a medical complaint or simply came to be present at the event and without consideration of their ultimate diagnosis. Additionally, we believe the selection bias effect would be limited by the large community turnout at these brigades which are treated as much as community events as medical encounters.

The results reported here suggest a relatively high prevalence of UTI in women of rural western Panama. There are numerous factors which could contribute to this clinical observation. The weather in Panama is tropical: hot and humid. This tropical climate coupled with poor sanitation and contaminated drinking water likely fosters a dangerous combination of dehydration and diarrheal illness. As the well accepted mechanism of UTI involves coliform bacteria from the host being transferred to a susceptible urethra, it would be reasonable to assume higher UTI rates where diarrheal illness is common and sanitation is poor. We believe that dehydration is likely to play a role as well, both by exacerbating urethral irritation and by limiting the likelihood of post-coital voiding thought to protect from UTI by cleansing the urethra. In addition to these environmental and climatic factors, lack of education regarding personal hygiene such as wiping from front to back is also likely to contribute to high UTI prevalence. It is unlikely that women in these areas have been provided with even a rudimentary understanding of the etiology of UTI or simple preventative techniques such as post-coital voiding and appropriate hygiene when toileting.

In this study we provide a systematic analysis supporting our clinical observations while on medical mission trips to Panama that women in rural western Panama appear to suffer an inordinately high rate of UTI. Further study is necessary to determine if there are other confounding variables that could affect a woman’s risk of developing UTI. Based upon the literature [9]–[13], these variables include socioeconomic status, educational level, parity, pregnant vs. non-pregnant state, age, and access to sanitation facilities. We intend to continue to study the prevalence of UTI in different provinces of Panama on future trips. In designing this future research, it is our intention that we expand our focus to include not just UTI prevalence but risk and effectiveness of prevention measures as well. We have developed an educational tool which we intend to not only distribute in areas where the prevalence of UTI is elevated, but also to evaluate the tool’s effectiveness in decreasing risk of the development of UTI. Finally, we plan to work with the Panamanian Ministry of Health to explore why their prevalence values are at odds with our own.

As the preventative techniques taught to women in the developed world are simple lifestyle adjustments and do not require advanced technology, resources or education, we believe that UTI may provide an accessible target for the development of health education programs aimed at reducing the prevalence of disease in the community and improving the quality of life for many women in low and middle income countries. On future trips, we intend to implement and assess the effectiveness of such programming in improving health literacy as well as reducing infection rates.
